# Diagnostic Parameters of Adenoviremia in Pediatric Stem Cell Transplant Recipients

**DOI:** 10.3389/fmicb.2019.00414

**Published:** 2019-02-22

**Authors:** Karin Kosulin, Herbert Pichler, Anita Lawitschka, René Geyeregger, Thomas Lion

**Affiliations:** ^1^Molecular Microbiology, Children's Cancer Research Institute, Vienna, Austria; ^2^Stem Cell Transplant Unit, St. Anna Children's Hospital, Vienna, Austria; ^3^Department of Pediatrics, Medical University of Vienna, Vienna, Austria

**Keywords:** human adenovirus, pediatric stem cell transplant recipients, viremia, area under the curve, non-relapse mortality

## Abstract

Despite recent progress in the diagnostic risk assessment of human adenovirus (HAdV) infections in immunocompromised patients, clinical complications mediated by these viruses continue contributing to significant morbidity and mortality, particularly in the pediatric hematopoietic allogeneic stem cell transplant (HSCT) setting. Current data highlight the importance of monitoring stool samples to assess the risk of invasive HAdV infections in children undergoing HSCT. The advent of novel, more effective antiviral treatment options might permit successful virus control even at the stage of systemic infection, thus increasing the interest in optimized HAdV monitoring in peripheral blood (PB). We have screened over 300 pediatric HCST recipients by serial monitoring of stool and PB specimens, and identified 31 cases of invasive HAdV infection by quantitative pan-adenovirus RQ-PCR analysis of consecutive PB specimens. The diagnostic parameters assessed included HAdV peak levels (PL) and the time-averaged area under the curve (AAUC) of virus copy numbers. The predictive value for patient outcome reflected by non-relapse and HAdV-related mortality was determined. The patients were assigned to quartiles based on their PL and AAUC, and the readouts were highly correlated (*p* < 0.0001). Non-relapse mortality in patients by AAUC quartile (lowest to highest) was 26, 50, 75, and 86%, respectively, and AAUC was strongly correlated with non-relapse mortality (*p* < 0.0001), while the association between PL and non-relapse mortality was less pronounced (*p* = 0.013). HAdV-related mortality was absent or very low in patients within the two lower quartiles of both PL and AAUC, and increased to ≥70% in the upper two quartiles. Despite the significant correlation of PL and AAUC with patient outcome, it is necessary to consider that the risk of non-relapse mortality even within the lowest quartile was still relatively high, and it might be difficult therefore to translate the results into differential treatment approaches. By contrast, the correlation with HAdV-related mortality might permit the identification of a low-risk patient subset. Nevertheless, the well-established correlation of HAdV shedding into the stool and intestinal expansion of the virus with the risk of invasive infection will expectedly remain an essential diagnostic parameter in the pediatric HSCT setting.

## Introduction

The incidence and severity of invasive HAdV infections in allogeneic HSCT recipients correlate with different factors including the level of immune system impairment, mediated in part by the conditioning regimen and the employment of strongly T-cell depleting strategies (Saif et al., 2015; Rustia et al., 2016). Preemptive treatment with cidofovir has been regarded as a standard of care in HSCT recipients displaying viremia (Matthes-Martin et al., 2012), but the response rates were suboptimal. The ability to control the infection by current therapeutic modalities, including also adoptive transfer of HAdV-specific T-cells, has been suggested to depend on timely onset of treatment (Lion et al., 2003; Feuchtinger et al., 2006; Geyeregger et al., 2014; Lion, 2014; Feucht et al., 2015). This notion has sparked the search for diagnostic parameters permitting early risk assessment of impending invasive infection and disseminated disease. In the pediatric HSCT setting, monitoring of serial stool specimens during the post-transplant period was shown to facilitate early HAdV detection, and expansion of viral loads in stool with peak levels exceeding 10E6 virus copies/g was demonstrated to confer a very high risk for systemic infection (Lion et al., 2010; Jeulin et al., 2011). In children undergoing HSCT, HAdV expansion in stool was reported to almost invariably precede invasive infection defined by the detection of viremia (Lion et al., 2010; Lion, 2014). The absolute level of virus copy numbers in stool identified as critical may vary depending on the specific techniques of quantitative analysis used by different laboratories (Lion et al., 2010; Jeulin et al., 2011; Legoff et al., 2017; Hum et al., 2018). In this regard, documentation of rapidly rising HAdV copy numbers in serial stool specimens may be a more universal parameter for risk assessment of imminent invasive infection (Lion, 2014). Recent data indicate that a proportion of children carry persistent HAdV in the gastrointestinal (GI) tract, with most prominent occurrence in the ileum (Kosulin et al., 2016c). The observed rates of HAdV persistence in the GI tract and reactivation of the virus post-transplant were similar, suggesting that intestinal virus persistence could be a key risk factor for ensuing HAdV-related complications after HSCT (Kosulin et al., 2016c). More recent findings revealed that intestinal HAdV shedding prior to transplantation confers a highly significant risk for early and rapid post-transplant expansion of the virus, leading to an elevated incidence of invasive infections associated with pronounced morbidity and mortality (Kosulin et al., 2018). The important role of stool monitoring is well-documented in the pediatric setting, but may be of lesser relevance in adult HSCT recipients in whom intestinal HAdV persistence and reactivation have not been documented to date. However, individual reports on transplant-related mortality in patients with adenoviremia suggest that these infections can also be severe and potentially life-threatening outside the pediatric setting (Ganzenmueller et al., 2011; Sive et al., 2012; Taniguchi et al., 2012; Lee et al., 2016; Ramsay et al., 2017). Due to the fact that the number of HAdV types has been steadily increasing as a result of homologous recombination events (Ismail et al., 2018), the current spectrum has expanded by almost forty newly recognized virus types over the last decade (http://hadvwg.gmu.edu/), reflecting the generation of recombinant viruses with potentially altered tissue tropism and pathogenicity. This implies that the methods used for diagnostic screening must be regularly controlled and adapted, if necessary, in order to ensure reliable detection of all known HAdV types (Kosulin et al., 2016a). Although many pediatric transplant centers have already adopted serial HAdV monitoring in stool samples as part of the routine diagnostic program, the decision to initiate antiviral treatment is still mostly based on detection of the virus in peripheral blood, with attempts to define thresholds of viral copy numbers deemed adequate for the onset of therapy (Teramura et al., 2004; Lindemans et al., 2010; Lee et al., 2013). Based on the observations that delayed treatment initiation by the available therapeutic modalities at the stage of invasive infection carries a considerable risk of failure, it has been suggested that, at least in the pediatric setting, it might be beneficial to start therapy guided by regular monitoring of stool specimens, before the HAdV infection becomes invasive (Lion, 2014; Hiwarkar et al., 2018). However, employment of the novel antiviral agent brincidofovir was shown in clinical studies to permit efficient HAdV elimination even in patients displaying viremia, and the efficacy of this drug was independent from immune reconstitution, in contrast to treatment with cidofovir (Florescu et al., 2012; Grimley et al., 2017; Hiwarkar et al., 2017). These clinical observations highlighted the need to establish improved diagnostic parameters based on the screening of peripheral blood samples. Serial monitoring of HAdV plasma levels has been commonly used to assess the response to therapy, but the possible role of the duration of adenoviremia reflecting the overall systemic exposure to the virus has not been investigated. A holistic measure that combines both parameters could permit improved monitoring of responses to different therapeutic interventions. To address this notion, we have evaluated the impact of peak HAdV levels and the area under the curve indicating virus plasma levels over time as diagnostic and prognostic parameters in pediatric HSCT recipients.

## Patients and Methods

### Patients

Serial samples including stool and peripheral blood specimens derived from 304 patients who were treated by allogeneic stem cell transplantation at the St. Anna Children's Hospital, Vienna, Austria, between the years 2000 and 2015, were investigated. Patient and transplant characteristics were published previously (Kosulin et al., 2018), and the most important parameters are displayed in Table 1. The samples were acquired within the routine HAdV screening program starting prior to conditioning, and thereafter at weekly intervals for a minimum of 100 days post-transplant. The present study was restricted to the analysis of test results derived from routine diagnostic analyses, with available written informed consents provided by individual patients and/or their parents. In line with previously published definitions (Lion et al., 2010), disseminated HAdV disease was diagnosed by the detection of multiple organ involvement (e.g., hepatitis, encephalitis, retinitis) in the presence of two or more HAdV-positive PCR assays in peripheral blood and other sites tested (e.g., cerebrospinal fluid, BAL, respiratory secretions, urine), in absence of other identifiable causes. All patients with fever, unexplained symptoms, or laboratory test abnormalities were carefully tested for infections and for GvHD. HAdV-associated death was defined as multiple organ failure in the presence of increasing or persisting adenoviral load in peripheral blood, in association with HAdV detection from multiple other sites, if pertinent.

**Table 1:** Patient characteristics.

**Pat**.	**Age**	**Underlying disease**	**Donor type**	**Conditioning**	**Engraftment (day post HSCT)**				**GvHD grade**
					**Leuko>200/μl**	**Leuko>1,000/μl**	**Gran>500/μl**	**T cells>300μl**	
1	1.3	Immunodeficiency	UD	RIC	22	47	99	68	0
2	12.2	Acute leukemia	UD	MA	23	26	24	268	0
3	1.7	Acute leukemia	UD	MA	16	20	21	75	1
4	8.2	Metabolic disease	UD	RIC	10	13	14	74	1
5	3.6	Immunodeficiency	UD	RIC	1	17	18	96	3
6	13.4	Lymphoma	UD	MA	12	14	24	n.a.	0
7	7.4	SAA	UD	RIC	17	20	21	64	4
8	5.1	DA	UD	RIC	13	16	15	n.a.	4
9	3.2	Immunodeficiency	MSD	RIC	21	35	41	115	0
10	16.6	Solid tumor	MMFD	RIC	11	13	13	n.a.	0
11	14.1	Acute leukemia	UD	MA	28	40	40	97	0
12	9.2	Acute leukemia	UD	MA	15	19	18	51	0
13	3.7	Solid tumor	MMFD	RIC	9	12	12	162	0
14	2.2	Solid tumor	MMFD	RIC	17	19	19	n.a.	0
15	10.5	Acute leukemia	UD	MA	16	19	19	n.a.	0
16	7.4	Chronic leukemia	UD	MA	17	32	32	n.a.	4
17	13.6	Lymphoma	UD	MA	28	32	34	111	1
18	9.4	Acute leukemia	MSD	MA	9	11	14	23	4
19	20.1	Acute leukemia	UD	RIC	24	30	29	n.a.	3
20	8.6	Acute leukemia	MMFD	MA	12	13	13	n.a.	0
21	15.7	Acute leukemia	UD	MA	22	24	24	53	3
22	0.6	Immunodeficiency	MMFD	RIC	15	20	20	357	0
23	17.5	Thalassemia	UD	RIC	13	16	15	92	0
24	7.2	Immunodeficiency	UD	RIC	n.a.	n.a.	n.a.	n.a.	0
25	5.6	Lymphoma	UD	MA	14	20	23	n.a.	0
26	8.8	Acute leukemia	UD	MA	17	19	19	n.a.	4
27	0.5	Immunodeficiency	UD	RIC	9	10	10	n.a.	0
28	18.4	Immunodeficiency	UD	RIC	13	n.a.	n.a.	n.a.	0
29	2.1	Acute leukemia	MMFD	MA	13	18	19	n.a.	4
30	17.5	Acute leukemia	UD	RIC	12	n.a.	21	n.a.	0
31	10.0	Acute leukemia	UD	MA	11	12	12	n.a.	0

*SAA, severe aplastic anemia; DA, dyserythropoietic anemia; UD, unrelated donor; MSD, matched sibling donor; MMFD, mismatched family donor; RIC, reduced intensity conditioning; MA, myeloablative conditioning; Leuko, leukocytes; Gran, granulocytes; GvHD, Graft vs. host disease; n.a., not achieved or patient died*.

Antiviral prophylaxis in the HSCT recipients included acyclovir 30 mg/kg per day intravenously from day −7 until day +28 for HSV-IgG positive patients and ganciclovir 10 mg/kg for CMV-IgG positive patients with CMV-IgG negative donors. Patients with a positive PCR test in peripheral blood for CMV or HAdV were regarded as eligible for preemptive antiviral treatment, regardless of the viral load. Treatment was initiated upon availability of PCR results, generally within 48–72 h after sampling. Patients with CMV-DNAemia received primary preemptive therapy with ganciclovir and, in case of persistent DNAemia, secondary preemptive treatment with cidofovir, until two consecutive negative results were obtained. Patients with adenovirus-DNAemia received preemptive treatment with cidofovir, sometimes in combination with ribavirin, if HAdV species C was present, until resolution of DNAemia. In cases of failed response to antivirals and absence of HAdV-specific T-cells, adoptive T-cell transfer with donor-derived HAdV-specific T cells was offered (Lion et al., 2010; Geyeregger et al., 2014).

### Isolation of Viral DNA

The QIAamp DNA Mini Kit (Qiagen) was used for extraction of DNA from peripheral blood (PB) specimens, and isolation of DNA from stool samples was done by employing the QIAamp DNA Stool Mini Kit (Qiagen), in line with recommendations provided by the manufacturer.

### RQ-PCR Analysis

For HAdV-screening, a pan-adenovirus real-time quantitative (RQ)-PCR assay was used involving the ABI 7500 Sequence Detectors (Thermo Fisher Scientific, Waltham, MA, USA), as specified in earlier reports (Ebner et al., 2005; Kosulin et al., 2016a,b). Samples revealing HAdV-positive test results by the screening assay indicated above were subjected to HAdV species identification by specific PCR tests (Lion et al., 2003). The limit of detection provided by the RQ-PCR tests employed was ten virus DNA copies per PCR reaction, and samples revealing ≥500 DNA copies/ml plasma were deemed HAdV-positive. Accordingly, the presence of adenoviremia was defined as the detectability of HAdV in peripheral blood at or above the indicated level. The HAdV virus burden over time was assessed by calculating the time-averaged area under the curve (AAUC) over 16 weeks from the onset of viremia with ≥1,000 copies/ml. For AAUC calculation, positive values below the lower limit of quantification and negative values within the 16-weeks observation time were also considered, and were assigned arbitrary values of 499 and 99, respectively.

### Statistics

The correlation between HAdV peak plasma levels and AAUC quartiles was assessed using the Pearson correlation supplemented by analysis of variance (ANOVA). Fine-Gray models were used to examine the association between AAUC (log_10_ copies/ml) and time to non-relapse mortality within 1 year of transplant, where relapse was considered as a competing risk and AAUC was included as a continuous covariate. HAdV-related mortality was analyzed similarly, with both relapse and non-HAdV associated deaths handled as competing risks. Hazard ratios (HR) and associated 95% confidence intervals (CI) were determined, to assess the relative risk for mortality in relation to a 1 log_10_ increase in AAUC (log_10_ copies/ml). Patients were divided into quartiles based on the PL and AAUC levels, and the median values for each quartile were determined. The quartiles of PL were approximated by logical grouping according to the thresholds indicated below, resulting in seven patients assigned to each of the first three quartiles and ten patients to the highest quartile. HRs and 95% CI were determined by comparing the median value of the lowest quartile vs. median values of the other three quartiles. All analyses were repeated to additionally examine the association of peak HAdV viremia and mortality. Pearson correlation and ANOVA were conducted using the GraphPad Prism 5 software, and Fine-Gray models were run using SAS version 9.4.

## Results

### Occurrence of Adenoviremia and Outcome

Within the cohort of 304 pediatric patients who underwent allogeneic HSCT at our center, screening by a universal RQ-PCR assay covering the entire spectrum of known HAdV species and types, 31 (10.2%) developed viremia, with HAdV detection of more than 10E3 virus copies/ml at a minimum of two subsequent time points. In nearly all patients who experienced invasive infection, the virus was detectable in stool at levels above the critical threshold of 10E6 copies/g prior to or at the onset of viremia. The only exceptions observed included a few patients in whom no stool data were available for several weeks prior to the onset of viremia (Table 2). The distribution of HAdV species detected in PB, displaying predominance of species C and A, corresponded to that detected in children with intestinal persistence of the virus (Kosulin et al., 2016c) (Table 2). Concurrent infections with other DNA viruses were frequent 28/31 (90%), most commonly with CMV, EBV and BKV (Table 2). During the observation time of 100 days post-transplant, first onset of viremia was detected between days 1 and 93, with a median on day 34 (Table 2). The overall mortality in patients with adenoviremia was 68% (21/31), and lethal outcome affected also six of nine patients who had received adoptive transfer of HAdV-specific T-cells in addition to standard treatment with cidofovir ± ribavirin (Table 2). The cause of death was attributable to HAdV-related disease in 39% (12/31), while the remaining patients succumbed to relapse of the underlying disease or other infectious or non-infectious complications (Table 2).

**Table 2:** Virus detection and outcome.

**Pat**.	**1st HAdV pos. in stool (day post HSCT)**	**>10E6 HAdV copies/g stool (day post HSCT)**	**1st HAdV pos. in PB (day post HSCT)**	**HAdV species**	**HAdV-specific T cell transfer**	**Cause of death**	**Co-infections**
1	−5	5	19	A		Encephalomyelitis	CMV
2	21	21	27	A		–	BKV, EBV, HSV-1
3	−14	−14	1	C		–	–
4	−9	4	26	A		Relapse	HHV-6
5	5	13	13	C		–	BKV, EBV, A. spp.
6	10	48	85	C		Relapse	BKV, HSV-1
7	−14	28	49	A	YES	–	BKV, PVB19, CMV, HSV-1, HHV-6,-7
8	17	17	19	A	YES	HAdV, CMV	CMV, *C. albicans*
9	−14	6	13	C		–	EBV, HSV1, HHV-6, NV
10	6	11	35	A	YES	SIRS	CMV, EBV
11	13	27	34	C		CNS-LPD, CMV	BKV, CMV, EBV
12	−14	61	72	C	YES	–	BKV, EBV, HHV-6
13	6	14	93	A	YES	–	CMV
14	14	14	21	A		–	EBV, HSV-1, HHV-7
15	n.a.	n.a.	40	C		SIRS	EBV
16	39	54	60	A		VOD	BKV, *C. albicans*
17	7	36	48	C		HAdV	BKV
18	34	34	48	A, C		HAdV	BKV, EBV, HHV-6
19	80	87	94	C		HAdV	CMV, *C. albicans*
20	−14	n.a.^*^	11	n.d.		HAdV	HHV-6, C.
21	53	67	67	C		HAdV	BKV, CMV, EBV, HHV-7, PVB19, *C. albicans*
22	−14	−14	1	E, F		–	BKV, InfA
23	5	12	50	C		–	CMV
24	−9	n.a.	1	C		HAdV	*A. fumigatus, C. albicans*
25	−7	65	85	C	YES	Relapse	EBV
26	61	68	75	C		Bacterial sepsis	BKV, CMV
27	−14	−14	1	A		HAdV	EBV
28	−2	5	12	C		HAdV	CMV, *C. albicans, C. glabrata*
29	−10	34	34	C	YES	HAdV	–
30	9	10	31	B, E	YES	HAdV	BKV, CMV, A. spp., *C. krusei*
31	17	20	28	C	YES	HAdV	BKV, CMV

CNS-LPD, central nervous system lymphoproliferative disorder; SIRS, Systemic Inflammatory Response Syndrome; VOD, veno-occlusive disease; BKV, BK virus; CMV, Cytomegalovirus; EBV, Epstein-Barr virus; HHV-6, human herpesvirus 6; HHV-7, human herpesvirus 7; HSV-1, herpes simplex virus 1; InfA, Influenza A virus; NV, Norovirus; PVB19, Parvovirus B19; C., Candida; A., Aspergillus; spp., species; n.a., no available analysis prior to viremia;

* *No quantitative detection of virus copies available; n.d., not determined*.

### Temporal Correlation Between Intestinal Adenovirus Reactivation and Onset of Viremia

Nearly all transplant recipients were screened for the presence of HAdV in serial stool specimens already prior to conditioning, and the virus copy numbers were assessed at weekly intervals at least until day 100 post-transplant. In line with our earlier observations (Lion et al., 2010), the onset of HAdV viremia was almost invariably preceded by detection of the virus in stool (Table 2). The presence of virus loads exceeding 10E6 copies per gram of stool, a threshold previously demonstrated to confer a very high risk of invasive infection, predated the onset of viremia by a median of 11 days (range 0–79; Table 2), corroborating the paramount importance of stool surveillance as a basis for timely risk assessment of impending HAdV viremia in the pediatric allogeneic HSCT setting.

### Peak Adenovirus Levels and Viral Burden Over Time (AAUC) in Peripheral Blood

**Figure 1:**
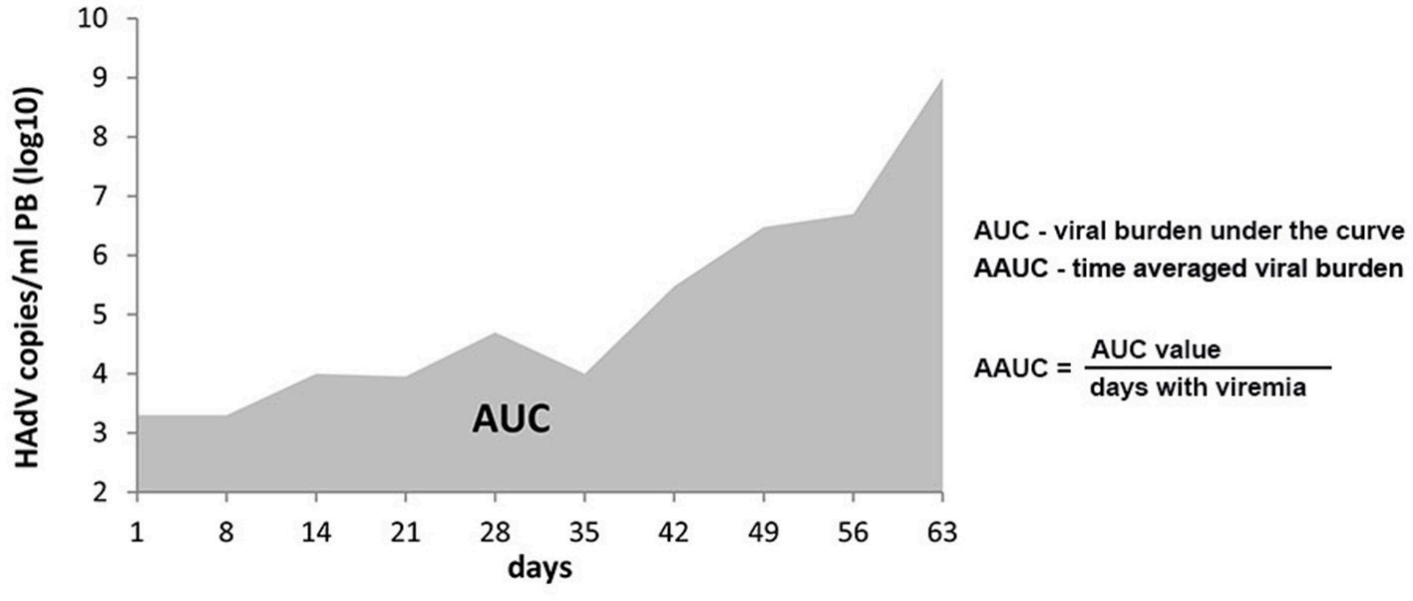
Area under the curve (AUC) and average time-dependent AUC (AAUC). An exemplary AUC of a patient who died from HAdV disease on day 63 after HSCT is shown. The formula underlying the calculation of AAUC is indicated. The denominator for AAUC is not just days with viremia; days alive and without viremia would contribute to averaging viral burden over time (i.e., through 16 weeks, if the patient is alive and available for follow-up at that time).

**Figure 2:**
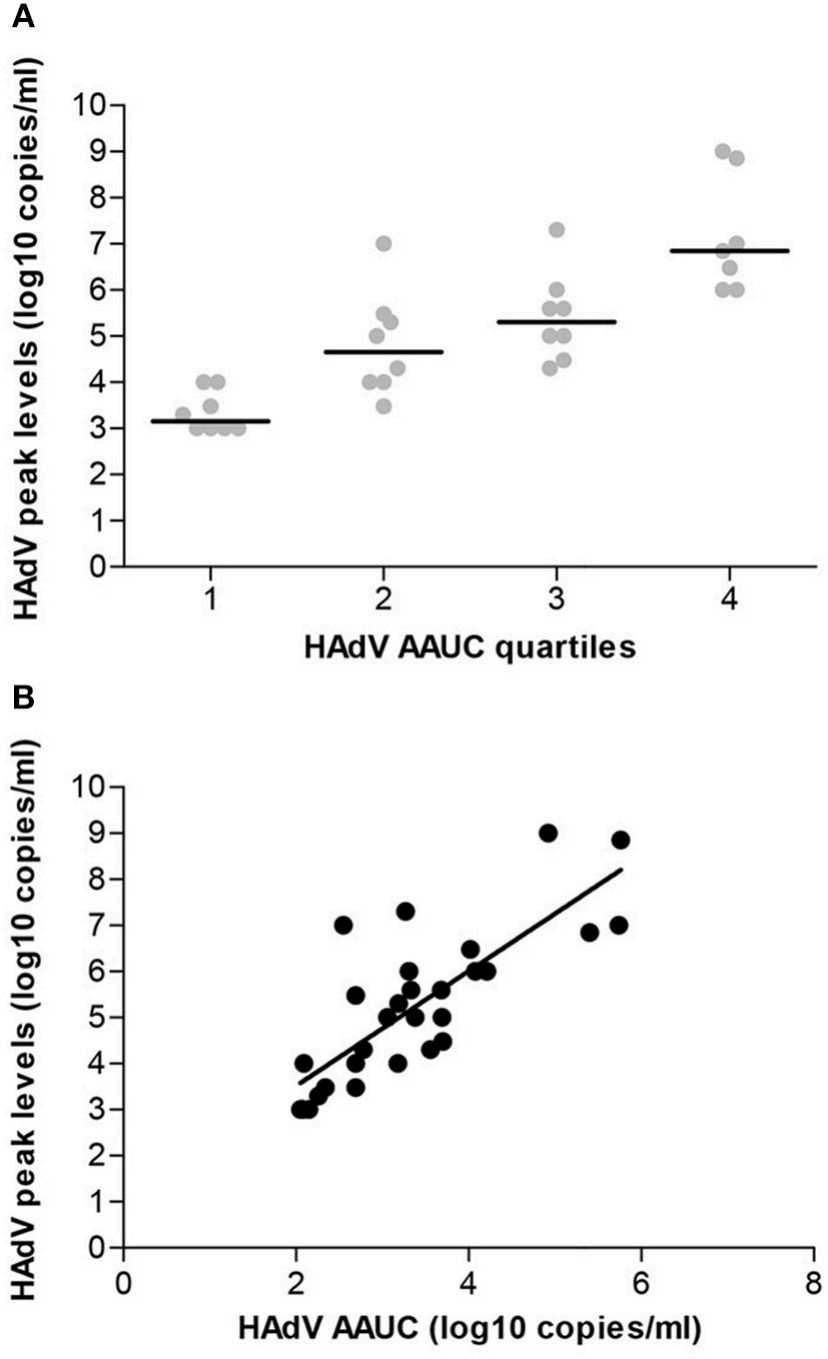
Correlation of HAdV peak levels in peripheral blood with viral burden over time. **(A)** The individual HAdV copy numbers assigned to AAUC quartiles are given (ANOVA variance analysis *p* < 0.0001). **(B)** The Pearson correlation shows a highly significant correlation between the AAUC values and the HAdV copy number peak values (*p* < 0.0001; *r* = 0.7977).

All patients with viremia had HAdV peak levels (PL) of at least 10E3 virus copies/ml PB, and the highest virus copy number observed in the cohort presented was 10E9/ml. The approximate assignment of patients to quartiles based on their peak HAdV copy numbers/ml PB revealed the following distribution: quartile 1, <10E4 (median 1 × 10E3); quartile 2, <10E5 (median 1 × 10E4); quartile 3, <10E6 (median 2 × 10E5); quartile 4, ≥10E6 (median 8.5 × 10E6). In order to determine the overall exposure of patients to the viral burden over time, the AAUC (log_10_ copies/ml) was calculated for each patient with viremia, as exemplified in Figure 1, yielding values between 2.05 and 5.76. The division of patients into quartiles according to their respective AAUC resulted in the following distribution: quartile 1: <2.50 (median 2.09); quartile 2: <3.25 (median 2.75); quartile 3: <4.0 (median 3.47); quartile 4: ≥4.0 (median 4.92). ANOVA showed a significant association of HAdV PL values with individual AAUC quartiles (*p* = 0.0001; Figure 2A), and direct comparison between PL and AAUC values in each patient revealed a significant correlation (*p* < 0.0001; R-squared 0.81; Figure 2B).

### Correlation of Peak Adenovirus Levels and Viral Burden Over Time (AAUC) With Patient Outcome

The HAdV peak viral load and AAUC were both correlated with non-relapse and HAdV-related mortality. An increasing rate of non-relapse mortality was observed with rising AAUC, revealing 25% in quartile 1, 50% in quartile 2, 75% in quartile 3, and 86% in quartile 4. The correlation was less clear for the quartiles of PL, with 43% in quartile 1, 57% in quartile 2, 43% in quartile 3, and 80% in quartile 4. HAdV AAUC was strongly associated with non-relapse mortality (*p* < 0.0001, HR 1.7, 95% CI 1.3–2.2) and HAdV-related mortality (*p* < 0.0001, HR 2.2, 95% CI 1.7–2.9). Hazard ratios relating HAdV AAUC quartiles are summarized in Table 3. Similarly, the PL of HAdV viremia was also associated with non-relapse mortality, albeit with less pronounced significance (*p* = 0.013, HR 1.3, 95% CI 1.1–1.6), and revealed a highly significant correlation with HAdV-related mortality (*p* = 0.0001, HR 1.7, 95% CI 1.3–2.2). The corresponding hazard ratios are summarized in Table 4.

**Table 3:** Adenovirus AAUC (log_10_ copies/mL) vs. mortality.

**Quartile (Range)**	**Non-relapse mortality HR (95% CI)**	**HAdV-related mortality HR (95% CI)**
4th (4.0–5.8)	4.4 (2.1–9.2)	9.2 (4.3–19.9)
3rd (3.3–3.7)	2.1 (1.5–3.0)	3.0 (2.0–4.3)
2nd (2.6–3.2)	1.4 (1.2–1.7)	1.7 (1.4–2.0)
1st (2.1–2.3)	Reference	Reference

**Table 4:** Peak adenovirus levels (log_10_ copies/mL) vs. mortality.

**Range**	**Non-relapse mortality HR (95% CI)**	**HAdV-related mortality HR (95% CI)**
≥6.0	2.8 (1.2–6.2)	7.3 (2.6–20.4)
5.0–<6.0	1.8 (1.1–2.9)	3.2 (1.8–5.9)
4.0–<5.0	1.3 (1.1–1.6)	1.7 (1.3–2.2)
<4.0	Reference	Reference

All patients with viremia received antiviral treatment with cidofovir (±ribavirin), and HAdV-specific T-cells derived from the original HSCT donor were provided to nine refractory patients (see Table 2). This immunotherapeutic intervention displayed good efficacy in three patients who cleared the infection upon T-cell therapy. However, four patients, including those who had received delayed adoptive transfer of HAdV-specific T-cells, had prolonged viremia with high HAdV burden, and ultimately succumbed to disseminated viral disease, while the remaining two T-cell recipients died from reasons unrelated to HAdV infection. Overall, non-relapse mortality was high in all patients who developed viremia (21/31; 68%), and could be attributed to probable HAdV disease in several instances (12/21; 57%) (Table 2).

## Discussion

In the present study, we have addressed the potential prognostic relevance of peak HAdV levels in peripheral blood as well as the viral burden over time as diagnostic parameters in pediatric HSCT recipients. These parameters appeared highly correlated (*p* < 0.0001), although some patients with high PL had lower HAdV AAUC due to the relatively short duration of viremia. However, the number of patients with discrepant assignment to PL vs. AAUC quartiles was too small to determine an impact on outcome. This partial discordance might conceivably be attributable to the effect of antiviral therapy including cidofovir (±ribavirin) and HAdV-specific T-cells.

The correlation of high HAdV load in PB with lethal outcome of the infection in the allogeneic HSCT setting, and consequently, the importance of quantitative monitoring of HAdV DNaemia, have been discussed previously (Ganzenmueller et al., 2011; Lion, 2014; Hiwarkar et al., 2017). Novel treatment options including particularly the antiviral agent brincidofovir, which has documented efficacy in patients with invasive HAdV infection (Florescu et al., 2012; Grimley et al., 2017; Hiwarkar et al., 2017; Ramsay et al., 2017; Lopez et al., 2018), highlight the need for additional PB-based diagnostic parameters permitting the assessment of viral response to treatment and prediction of outcome. The total adenoviral burden over a fixed time period, calculated as HAdV AAUC, captures both peak and duration of viremia. Moreover, the variability in follow-up time inferred by early mortality is controlled by the AAUC. This parameter is an established virological endpoint for quantifying the course and severity of disease in acute lytic viral infections, and has been used previously as a primary endpoint in clinical studies of investigational antivirals (Pulido et al., 2004; Lamarca et al., 2006; DeVincenzo et al., 2014; Vegvari et al., 2016). The employment of AAUC in the present study revealed a clear correlation with patient outcome based on non-relapse mortality, which displayed a continuous increase along the quartiles (Figure 2A). The risk of non-relapse mortality was ≥50% in patients assigned to AAUC quartiles 2–4, but it is necessary to consider that the proportion of lethal outcomes within quartile 1 was still as high as 25%. The same principal consideration applies to the peak HAdV levels in PB, where non-relapse mortality was above 40%, regardless of the PL quartile. Despite the more or less pronounced differences between individual AAUC and PL quartiles, these observations indicate that non-relapse mortality was high in all patients who had developed viremia. The number of viremic patients included in the present study is certainly limited, and interpretation of the data must therefore be performed with great caution. Nevertheless, the findings suggest that the onset of viremia at any level may represent a high-risk situation requiring improved clinical management to decrease the mortality rate. In fact, based on the observations presented, differential treatment approaches based on the assignment to different PL or AAUC quartiles would not seem pertinent, but measures that can reduce peak viral load and AAUC are likely to improve outcomes. The recently reported superior efficacy of brincidofovir in comparison to cidofovir in pediatric HSCT recipients with HAdV viremia (Hiwarkar et al., 2017) might, at least in part, be attributable to the fact that oral brincidofovir delivers the antiviral effect directly to the GI tract, which appears to be the primary site of viral replication in this setting.

Invasive HAdV infections represented by viremia occur primarily by representatives of the species A, B, and C, and, in line with published data, the latter species was also most prevalent in viremic patients in the present study. This observation is supported by the predominance of HAdV species C in children with persistence of the virus in the gastrointestinal tract, which is the main site of reactivation and expansion in the pediatric allogeneic HSCT setting, as demonstrated previously (Kosulin et al., 2016c). Other HAdV species (D-G) are far less frequently detected in peripheral blood, and mostly occur in combination with one of the common species (Lion, 2014). There is no evidence, however, that the presence of any particular HAdV species or type in peripheral blood, either alone or in combination, has any relevant impact on the response to antiviral treatment. Neither cidofovir nor brincidofovir reveal any differences in their efficacy against various HAdV species. Only ribavirin, which has occasionally been used in addition to cidofovir, may only be effective, if at all in the clinical setting, against HAdV species C (Lion, 2014). Although HAdV-specific T-cells might conceivably display differential efficacy depending on the species present, there are currently no data supporting the notion that the HAdV species present could affect outcome of the disease.

Current data support the notion that the prevention of invasive HAdV infection should be the paramount aim in order to reduce transplant-related mortality in HSCT recipients. In the pediatric transplant setting, the role of HAdV reactivation and expansion in the intestinal tract has been clearly established as a pre-eminent risk factor for ensuing invasive infection (Lion et al., 2010; Jeulin et al., 2011; Legoff et al., 2017; Hum et al., 2018; Kosulin et al., 2018), and the expected clinical availability of effective antiviral treatment options displaying relatively low toxicity might greatly facilitate stool-guided preemptive therapy (Hiwarkar et al., 2018). The observation that serial HAdV monitoring of stool specimens in children undergoing allogeneic HSCT permits reliable assessment of the risk for invasive HAdV infection is well-documented (Lion et al., 2010; Lion, 2014; Hiwarkar et al., 2018). It was shown that the median time span between the documentation of HAdV copy numbers in stool exceeding the critical threshold of 10E6/g and the onset of viremia was 11 days (Lion et al., 2010; Lion, 2014), thereby providing a rational window of opportunity for early initiation of treatment, in an attempt to prevent systemic infection and disseminated disease. The proposed algorithm for HAdV monitoring and treatment in the pediatric HSCT setting based on serial monitoring of stool samples (Lion, 2014) may remain relevant even if more effective treatment options permitting control of the disease at the stage of viremia become readily available, because early containment of the infection at the pre-invasive stage could be perceived as a preferable strategy with regard to the expected outcome. Besides the considerations pertaining to the importance of preventing invasive infection, the parameters investigated in the present study indicated an intriguing correlation with HAdV-related mortality, which was very low in the AAUC quartiles 1 and 2, and in the PL quartile 1, respectively, including a single patient who has died from probable HAdV infection. Hence, these findings would indicate that virus levels in PB below 10E4 copies/ml and AAUC levels below 3.2 confer a low probability of succumbing to the viral infection, thereby providing diagnostic markers of potential prognostic relevance.

It is necessary to bear in mind, however, that the commonly used definitions of probable or proven HAdV disease may not be universally employed (Lion et al., 2010; Matthes-Martin et al., 2012), and unequivocal attribution of the cause of death to HAdV may be difficult to establish without autopsy and proof of (multiple) organ involvement. Since in severely immunocompromised transplant recipients, co-infections with multiple (viral and other) pathogens are not uncommon, as indicated in Table 2, the assignment of the cause of death is supported, in part, by the clinical findings compatible with specific infections and, in part, by the diagnostic monitoring of individual pathogens in the presence of concomitant infections. In case of HAdV-related mortality, which is often associated with multi-organ failure, lethal outcome is generally preceded by high or steadily increasing copy numbers of the virus in serial peripheral blood specimens (Lion et al., 2003; Lion, 2014). If other concomitant infections (including particularly CMV, which can cause similar clinical symptoms), are detectable at low or declining copy numbers during HAdV predominance, the latter virus may be regarded as the main culprit. Nevertheless, the partially discrepant observations between non-relapse and HAdV-related mortality in the present study may indicate that the former term, which also encompasses graft-vs.-host disease, treatment-related toxicity and other infections, may overestimate the actual contribution of HAdV to lethal outcome. In this regard, it would be of interest to employ more extensive diagnostics to facilitate reliable assessment of the actual pathogenetic role of HAdV in organ damage and mortality. Despite the recent insights and the progress in the molecular monitoring of invasive HAdV infections in the HSCT setting, optimal diagnostic surveillance remains a challenging task.

## Author Contributions

KK analyzed and interpreted the data, designed the study. HP and AL provided patient samples and were involved in data collection. RG was involved in data analysis and interpretation. TL designed the study, was involved in data interpretation and manuscript preparation.

### Conflict of Interest Statement

TL Chimerix-consultancy and honoraria. The remaining authors declare that the research was conducted in the absence of any commercial or financial relationships that could be construed as a potential conflict of interest.
